# Ovothiol ensures the correct developmental programme of the sea urchin *Paracentrotus lividus* embryo

**DOI:** 10.1098/rsob.210262

**Published:** 2022-01-19

**Authors:** Alfonsina Milito, Maria Cocurullo, Alfredo Columbro, Simona Nonnis, Gabriella Tedeschi, Immacolata Castellano, Maria Ina Arnone, Anna Palumbo

**Affiliations:** ^1^ Department of Biology and Evolution of Marine Organisms, Stazione Zoologica Anton Dohrn, Naples, Italy; ^2^ Department of Molecular Genetics, Centre for Research in Agricultural Genomics, Barcelona, Spain; ^3^ Department of Veterinary Medicine (DIMEVET), Università degli Studi di Milano, Milan, Italy; ^4^ CRC ‘Innovation for Well-Being and Environment’ (I-WE), Università degli Studi di Milano, Milan, Italy; ^5^ Department of Molecular Medicine and Medical Biotechnology, University of Naples Federico II, Naples, Italy

**Keywords:** ovothiol, oxidative stress, cell proliferation, sea urchin, embryonic development, marine organisms

## Abstract

Ovothiols are π-methyl-5-thiohistidines produced in great amounts in sea urchin eggs, where they can act as protective agents against the oxidative burst at fertilization and environmental stressors during development. Here we examined the biological relevance of ovothiol during the embryogenesis of the sea urchin *Paracentrotus lividus* by assessing the localization of the key biosynthetic enzyme OvoA, both at transcript and protein level, and perturbing its protein translation by morpholino antisense oligonucleotide-mediated knockdown experiments. In addition, we explored the possible involvement of ovothiol in the inflammatory response by assessing *ovoA* gene expression and protein localization following exposure to bacterial lipopolysaccharide. The results of the present study suggest that ovothiol may be a key regulator of cell proliferation in early developing embryos. Moreover, the localization of OvoA in key larval cells and tissues, in control and inflammatory conditions, suggests that ovothiol may ensure larval skeleton formation and mediate inflammatory processes triggered by bacterial infection. This work significantly contributes to the understanding of the biological function of ovothiols in marine organisms, and may provide new inspiration for the identification of the biological activities of ovothiols in humans, considering the pharmacological potential of these molecules.

## Graphical abstract

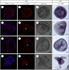

## 1. Introduction

Embryonic development represents an extremely sensitive and delicate stage of life for all living organisms, especially for those with external fertilization, such as sea urchins. Indeed, in the marine ecosystem, sea urchin adults and embryos are constantly exposed to a variety of environmental pressures, including both intrinsic and extrinsic/anthropogenic factors causing oxidative and nitrosative stress [1–7]. Nevertheless, they have acquired the ability, to some extent, to maintain homeostasis in an adverse environment, through the evolution of a ‘chemical defensome’, an integrated network of gene families and pathways, involved in the protection and repair from damage [8]. Moreover, since embryos lack specific tissues and organs for defence, all cells possess protective and antioxidant systems, and the dysregulation of such mechanisms can alter development, causing teratogenesis and lethality. Among the key biological processes underpinning embryonic development is programmed cell death (PCD). Apoptosis in particular represents a crucial mechanism of PCD, essential for the development and re-modelling of cell and organ structures in all living organisms, including sea urchins [9]. To support early development, eggs accumulate, during oogenesis, some antioxidant natural molecules, like tocopherols, ascorbic acid, carotenoids, glutathione and ovothiol [10–15].

Ovothiols in particular are π-methyl-5-thiohistidines, which are present in great amounts, compared to other intracellular thiols such as glutathione, in the eggs of sea urchins and other marine invertebrates [14,15]. They are also produced by some protists and proteobacteria, and their chemical structure confers them unique redox properties [16,17]. Since their discovery in the early 1980s, studies addressing the biological role of these molecules have been still limited. Recently, ovothiols have been reported to protect marine organisms, such as sea urchins, anemones, fish and mussels, from environmental stressors [18–21]. Moreover, they have been proposed to protect pathogenic parasites from macrophage-triggered oxidative stress during host infection [22], and microalgae from light-induced stress [23,24]. However, the role of these molecules does not seem to be restricted to a protective function. For example, they can act as mating pheromones in marine polychaetes, inducing egg release during sexual reproduction [25,26], and they are employed as a hunting strategy by cone snails, whose venom contains an ovothiol-derived structure (conazolium), used to mimic the natural pheromones of their preys (i.e. polychaetes) [27].

In sea urchins, ovothiols have been reported to control the H_2_O_2_ toxicity in the oocytes during the oxidative burst at fertilization [28] and to protect developing embryos from environmental cues, such as heavy metals and marine toxins [18]. Indeed, being broadcast spawners, sea urchins release gametes in the seawater column, where, upon egg–sperm interaction and fertilization, the developing embryos enter the plankton community and may cope with several stressful agents. These can induce the formation of reactive oxygen species (ROS), finally triggering the expression of the gene encoding the key enzyme involved in ovothiol biosynthesis, the 5-histidylcysteine sulfoxide synthase OvoA, and the consequent production of ovothiol molecules, which may contribute to the stress defence of the embryo [18]. Despite the increasing interest around these molecules, including their pleiotropic activities in humans [29–35] and the peculiar evolutionary history and widespread distribution of the biosynthetic enzymes [36,37], no functional studies have been performed so far to address the biological role of ovothiol.

Our hypothesis is that the extremely high abundance of ovothiols in the eggs and early embryos may underline a key function of these molecules to ensure fertilization and correct development. Here we carried out a comprehensive study addressing the biological relevance of ovothiol biosynthesis during the embryonic development of the sea urchin *Paracentrotus lividus*. To this end, we assessed the spatial expression of OvoA at different developmental stages, from unfertilized eggs to plutei larvae, at both transcript and protein levels, by *in situ* hybridization and immunohistochemistry (IHC) experiments. We also followed the temporal expression of OvoA during development, through western blot (WB) analyses. More importantly, we performed OvoA-targeted perturbation experiments through zygotic injection of specifically designed morpholino antisense oligonucleotides (MASO). The embryo phenotype caused by MASO-induced downregulation of ovoA translation was analysed by biochemical assays and confocal imaging. Finally, to investigate a possible role in the defence mechanisms of the larva, we exposed sea urchin plutei to a microbial lipopolysaccharide (LPS) mimicking bacterial infection and analysed effects on *ovoA* gene expression and OvoA enzyme immunolocalization. This is the first functional study assessing the localization of OvoA during the embryogenesis of an ovothiol-producing organism and the phenotype induced by OvoA downregulation.

## 2. Material and methods

### 2.1. Sea urchin sampling

*Paracentrotus lividus* (Lamarck, 1816) sea urchins were collected during the breeding season by scuba divers in the Gulf of Naples from a location not privately owned nor protected in any way.

### 2.2. Embryo culturing and treatments

Sea urchins were transported in an insulated box to the laboratory within 1 h after collection, kept in tanks with circulating seawater at a density of one animal per 5 litres and fed every 3 days with fresh macroalgae (*Ulva* sp.). The animals were acclimated for a minimum of 10 days before the experiments, during which very rare spontaneous spawning or mortalities were observed. Gamete spawning was induced by the injection of a 0.5 M KCl solution through the peribuccal membrane of the animals. Concentrated sperm was collected dry from at least three different males, mixed and kept undiluted at 4°C until use. Eggs from individual females were washed three times with 0.22 µm filtered seawater (FSW) and fertilized at a density of 150 eggs ml^−1^ with a few drops of diluted sperm (1 : 1000). Fertilization success was approximately 90%. For *in situ* hybridization and IHC experiments on pre-hatching developmental stages, eggs were fertilized in the presence of para-aminobenzoic acid (1 mmol l^−1^) to prevent hardening of the fertilization membrane, then removed by passing the fertilized eggs through a 70 µm mesh filter. Embryos were allowed to develop at 18 ± 2°C in a controlled temperature chamber at a 12 : 12 light : dark photoperiod cycle. All the experiments were performed at least in triplicate. For LPS treatments, sea urchin embryos were reared in 6-well plates (about 400 embryos per well), each well was filled with a total of 4 ml of FSW. We performed two biological replicates. Gametes from two females (F) and two males (M) were fertilized as follows: F1 × M1, F2 × M2. After checking that both cultures were properly dividing, they were mixed to obtain a mixed batch in order to increase genotypic diversity of offspring. Pluteus larvae at 48 h post fertilization (hpf) were treated with FSW (untreated controls), or 10 µg ml^−1^, 50 µg ml^−1^ or 100 µg ml^−1^ of LPS (O55:B5, Sigma). Stock LPS solutions were prepared in Milli-Q water at 5 mg ml^−1^ concentration. Larvae were developed as reported above and collected at three time points: 1, 2 and 4 h of treatment. Larvae were then used for phenotypic observation (using a Zeiss Axio Imager M1 microscope), IHC and RNA extraction.

### 2.3. Probe synthesis and *in situ* hybridization

Total RNA was extracted from about 1500 embryos (5 hpf blastulae) using RNAqueous-Microkit (Ambion), and 600 ng were retrotranscribed with iScriptTM cDNA Synthesis kit (Biorad), following the manufacturer's instructions. 1 µl of total cDNA was used to amplify an ovoA fragment (amplicone size: 974 bp) by PCR using the following primers: forward 5′-CATCCGTCCTCATCCGTCAG-3′; reverse 5′-CCTAACTGGCACGTCTTGGT-3′. The obtained PCR fragment was then cloned into the pGEM-T Easy Vector (Promega) and the plasmid fraction was purified by GenElute HP Plasmid Miniprep Kit (Sigma) and linearized by PCR. The PCR product was purified by QIAquick PCR Purification Kit (Qiagen) and used as a template for RNA labelling with digoxigenin-UTP by *in vitro* transcription with SP6 and T7 polymerases, to obtain antisense and sense probes respectively (electronic supplementary material, figure S1). Probes were finally purified by gel filtration chromatography on Sephadex G-50 columns and quantified by assessing the absorbance at 260 nm (ND-1000 Spectrophotometer; NanoDrop Technologies, Wilmington, DE, USA). *In situ* hybridization experiments were performed according to the protocol described by Andrikou *et al*. [38]. Briefly, embryos were fixed in fixative solution (4% paraformaldehyde, 0.1 M MOPS pH 7, 0.5 M NaCl, DEPC water) for 1 h at room temperature (RT), washed thrice in MOPS buffer (0.1 M MOPS pH 7, 0.5 M NaCl, 0.1% tween-20, DEPC water) and stored at −20°C in 70% ethanol in DEPC water until use. After three washes in MOPS buffer, the embryos were incubated in hybridization buffer (MOPS buffer supplemented with 70% formamide, 1 mg ml^−1^ BSA and 1 µl tRNA) for 3 h at 50°C and treated with antisense probe (0.1 ng µl^−1^) for one week at the same temperature. Then, samples were rinsed in post-hybridization buffer (MOPS buffer supplemented with 70% formamide, and 1 mg ml^−1^ BSA) for 3 h at 50°C and washed five times in MOPS buffer. For fluorescent *in situ* hybridization (FISH), embryos were blocked with Perkin-Elmer (PE) blocking reagent for 30 min at RT and then incubated with anti-digoxigenin antibodies conjugated to horseradish peroxidase (anti DIG-POD, Roche) 1 : 1000 in PE reagent overnight at 4°C. Following five washes in MOPS buffer, samples were incubated in amplification diluent (AD, 1.6 µl 30% H_2_O_2_ in 10 ml Tris-buffered saline, TBS 1×, 50 mM Tris–HCl, pH 7.6, 150 mM NaCl) for 30 min at RT and then treated with Cyanin 5 (Cy5, FP1171, Perkin Elmer) 1 : 400 in AD for 30 min at RT in dark. 4′,6-diamidine-2′-phenylindole dihydrochloride (DAPI) was added to the samples (1 : 5000; final concentration 0.2 µg ml^−1^) and embryos were visualized at the confocal microscope (Zeiss LSM 700). For chromogenic *in situ* hybridization (CISH), samples were washed four times in alkaline phosphatase (AP) buffer (0.1 M Tris–HCl, pH 7.6, 2.5 mM MgCl_2_, 0.1 M NaCl) and then twice in AP buffer supplemented with 0.2% tween-20 and 1 mM levamisole. Incubation was carried out in dye solution composed of 10% dimethylformamide, 100 mM Tris–HCl pH 9.5, 50 mM MgCl_2_, 0.1 M NaCl, 1 mM levamisole and the two substrates for alkaline phosphatase: 0.3 mg ml^−1^ nitro-blue tetrazolium chloride (NBT) and 0.2 mg ml^−1^ 5-bromo-4-chloro-3'-indoly phosphate p-toluidine (BCIP). Coloration was carried out overnight at RT, checking under optical microscope until the desired intensity was reached. The reaction was stopped with 50 mM EDTA in TBS 0.1% tween-20 (TBS-T). Samples were washed twice in TBS-T, rinsed in glycerol 30% in MOPS buffer and finally visualized on glass slides under the optical microscope (Zeiss Axio Imager M1). Negative controls were performed on the same batch of animals and in the same conditions using sense probe. For FISH and CISH experiments, about 50 embryos/larvae were visualized under the microscope for each developmental stage.

### 2.4. OvoA-MASO and synthetic mRNA injection

The ovoA-MASO, targeting the ovoA translation initiation site, was newly designed and acquired from Gene Tools (Corvallis) (5′-TTCGAGGCTCAGTTCCGTTGCCATG-3′). For each experiment around 200 fertilized eggs were injected with approximately 2–4 pl of 0.3 mM ovoA-MASO solution containing 0.12 M KCl. Each experiment was repeated at least thrice and included negative control embryos, injected with 0.3 mM of the standard control morpholino (GeneTools). The injection of the standard control morpholino did not cause any effect on the development of embryos. For rescue experiments, the complete ovoA CDS was *in vitro* synthesized as follows. Total RNA was extracted from about 1500 *P. lividus* unfertilized eggs using RNAqueous-Microkit (Ambion), and 1 µg was retrotranscribed with Super ScriptTM IV VILOTM Master Mix (Invitrogen), following the manufacturer's instructions. 1 µl of total cDNA was used to amplify the ovoA CDS, starting from the second methionine (amplicone size: 2235 bp) by PCR using the following primers: OvoA_BgIII 5′-GAAGATCTATGACACCCTGTGACCTCTC-3′; OvoA_EcoRI 5′-CGGAATTCTTACTGGGCCTCAGCTCTGG-3′. The PCR reaction was loaded on 1% agarose gel (electronic supplementary material, figure S2A) and the PCR product was purified from the gel using the GeneElute Gel Extraction Kit (Sigma) according to the kit's instructions. The purified PCR fragment was then digested with BgIII and EcoRI and cloned into pBlueScript pRN3 Plasmid [39]. The plasmid fraction was purified by GenElute HP Plasmid Miniprep Kit (Sigma) and linearized by Sfil digestion. Capped synthetic mRNA was obtained by *in vitro* transcription using mMESSAGE mMACHINE kit (Ambion), followed by DNAse treatment and LiCl precipitation, according to the kit's instructions. The mRNA integrity was checked by gel electrophoresis (electronic supplementary material, figure S2B) and mRNA concentration was quantified by assessing the absorbance at 260 nm (ND-1000 Spectrophotometer; NanoDrop Technologies, Wilmington, DE, USA). For rescue experiments, fertilized *P. lividus* eggs were injected with a working solution containing 0.3 mM ovoA-MASO, 0.75 µg µl^−1^ synthetic ovoA mRNA and 0.12 M KCl. The eventual toxicity of mRNA was evaluated by injecting the eggs with a solution containing 0.12 mM KCl and 0.75 µg µl^−1^ of ovoA mRNA, in absence of ovoA-MASO.

### 2.5. 5-ethynyl-2′-deoxyuridine and TUNEL assay

For detection of actively replicating DNA, live embryos were pre-incubated with 10 µM EdU for 2 h. Fixation and 5-ethynyl-2’-deoxyuridine (EdU) assay were performed according to the protocol described by Wood *et al*. [40]. For the terminal deoxynucleotidyl transferase dUTP nick end labelling (TUNEL) assay, 18–20 hpf embryos, both control and ovoA-MASO injected were fixed with 4% paraformaldehyde in FSW for 1 h. After three washes in PBS 0.1% tween-20 (PBS-T), embryos were treated with ice-cold methanol for 2 min, then rinsed twice in PBS for and in PBS-T two more times. For positive control, an aliquot of the control uninjected embryos was incubated with 1 U ml^−1^ DNase (Roche) at 37°C for 30 min and then washed twice with milliQ water. The assay was performed using Click-iT Plus TUNEL Assay (Invitrogen) according to the kit's instructions. Briefly, embryos were first rinsed in 1× TdT reaction buffer for 10 min at 37°C, and then incubated for 1 h with TdT reaction mix (1× TdT reaction buffer, 1× EdUTP nucleotide mixture, 0.6 U µl^−1^ TdT enzyme). After one wash in milliQ water, embryos were rinsed twice in 1% BSA in PBS-T and then washed twice with PBS. Embryos were then incubated for 30 min at 37°C with Click-iT reaction mix, containing 1× Click-iT Plus TUNEL Reaction Buffer Additive and Supermix (1x Click-iT Plus TUNEL Reaction Buffer, Copper Protectant, Alexa Fluor 488 picolyl azide). Following two incubations with 1% BSA in PBS, samples were washed thrice in PBS and incubated with Hoechst 33342 to stain the nuclei (1 : 2000 in PBS; final concentration 5 µg ml^−1^). Embryos were finally washed twice in PBS and visualized under the confocal microscope (Zeiss LSM 700).

### 2.6. Antibody production, IHC and WB analyses

In order to obtain a specific antibody for *P. lividus* OvoA protein (*Pl*OvoA), we selected three antigenic peptides based on its amino acidic sequence (ID: AMM72581.1) and structural model [18]. The selected peptides were: P1 (KTEDAIYKAPDRLRLC; 52–67 aa) and P2 (PDQNQNSSQYRYRSC; 313–327 aa), both exposed on the surface of the protein, and P4 (KSAEELLSKKQKVFYC; 609–624 aa), in the C-terminal region. The mix of the three synthetic ovalbumin conjugated peptides were used to immunize a rabbit, producing immune sera, from which the specific IgGs were purified (Primm, www.primm.it). The *Pl*OvoA specific antibody was used for all IHC and WB analyses. Pre-immune serum was provided by Primm, from which we purified unspecific IgGs by affinity chromatography using protein A agarose (Sigma) according to standard procedures. The pre-immune IgGs were used for negative controls both in IHC and WB analyses. For IHC, different *P. lividus* developmental stages, from unfertilized eggs to gastrula stages, were fixed in 2% paraformaldehyde in FSW for 10 min at RT and then treated with ice-cold methanol for 1 min. Plutei larvae were fixed in 4% paraformaldehyde in FSW for 15 min at RT, without ice-cold methanol treatment. After multiple washes in PBS-T, embryos were blocked in 4% sheep serum in PBS-T for 1 h at RT and incubated overnight at 4°C with the anti-OvoA IgGs (dilution 1 : 100, final concentration 5 µg ml^−1^) in 4% sheep serum in PBS-T. Pre-immune IgGs were used at the same protein concentration as negative control. After multiple washes in PBS-T, embryos/larvae were incubated with CF555 Goat Anti-Rabbit IgGs (H + L) (cat. 20033, Biotium, CA, USA) for 2 h in dark at RT (dilution 1 : 1000) in 4% sheep serum, then rinsed in PBS-T, added with DAPI (1 : 5000; final concentration 0.2 µg ml^−1^) to stain the nuclei. About 50 embryos/larvae for each developmental stage were finally visualized under the confocal microscope (Zeiss LSM 700). For IHC experiments on LPS-treated embryos and relative untreated controls, immunostaining was performed according to Perillo *et al*. [41]. OvoA antibody was diluted 1 : 100 and the incubation was performed at 4°C overnight, while Msp130 (6a9) [42] was diluted 1 : 50 and embryos were incubated at 37°C for 1 h and 30 min. AlexaFluor secondary antibodies (488 rabbit and 555 mouse) were used at 1 : 1000 at RT for 1 h. Samples were imaged using a Zeiss LSM700 confocal microscope equipped with a 25× water immersion objective, and keeping gain and laser power constant in each experiment. Images were analysed using ImageJ and ZEN 3.1 (blue edition). Brightness/contrast was adjusted only on the double immunostainings with Msp130, on the zoomed details of the pictures. On the contrary, no corrections have been applied to pictures of the whole larva, in order to allow the comparison of the staining under different treatments. For WB, 200 ml of culture (30 000 embryos in total) for each developmental stage (from unfertilized eggs to larval stages) were centrifuged at 1500 rpm for 10 min at 4°C in a swing-out rotor centrifuge. The collected pellets were combined, washed once in PBS, centrifuged again in the same conditions and the resulting pellets were weighed, rapidly freezed in liquid N_2_ and kept at −80°C until use. Pellets were dounce-homogenized on ice in PBS 0.5% Triton X-100, pH 7.4 [43], supplemented with proteases and phosphatases inhibitors (1 mM NaVO_4_, 1 mM NaF, 1 mM PMSF, cocktail of phosphatases and proteases inhibitors). A protein amount corresponding to 20 µg was separated by sodium dodecyl sulfate-polyacrylamide gel electrophoresis (SDS-PAGE) on 12% gels and transferred to PVDF membranes. Membranes were incubated with 5% non-fat dried milk (nfdmilk) blocking solution for 1 h and then with anti-OvoA IgGs (dilution 1 : 500, final concentration 1 µg ml^−1^s) or anti-α actin (SIGMA, 1 : 5000) overnight at 4°C. After multiple washes in PBS-T, membranes were incubated with horseradish peroxidase-conjugated goat anti-rabbit IgGs (Santa Cruz Biotechnology, 1 : 5000) in 5% nfdmilk for 1 h at RT. Protein bands were visualized on Hyperfilm-ECL films using ECL western blotting detection reagents (GE Healthcare). Optical density (OD) of immune-positive bands was quantified through ImageJ software. Data were presented as OvoA/actin OD ratios mean ± standard deviation and analysed by Kruskal–Wallis with a Dunn's *post hoc* test (*n* = 3 biological triplicate) using PAST software package, v. 4.03 [44]. Data with *p*-value < 0.05 were considered significant.

### 2.7. RNA extraction, cDNA synthesis and real time qPCR

Around 400 larvae per treatment were collected for RNA extraction in a 1.5 ml tube and centrifuged at maximum speed for 5 min. The pellet was resuspended in Lysis Buffer and vigorously vortexed to lyse the cells. The samples were fast frozen in liquid nitrogen and stored at −80°C until usage. RNA extraction was performed using RNAqueous-Micro Kit from ThermoFisher following manufacturer's instruction. cDNA has been synthesized in a 20 µl reaction from 200 ng of total RNA using the SuperScript VILO cDNA synthesis kit (Invitrogen) and stored at −20°C until use. For Real-Time qPCR, 10 µl of reaction was prepared as follows: 5 µl of SYBR Green reagent, 0.7 µl of forward and reverse primer mix (final concentration 0.7 µM each) and 1 µl of cDNA (diluted 1 : 10). Reactions were performed in four technical replicates using ViiA 7 Real Time PCR. The following primers were used for OvoA: forward AGGTCAGCATGGACATAGCC, reverse CCTCAGCCGACTTCAAGAAC. All primer pairs were validated by QPCR against a negative (water) control. Obtained data were analysed using REST. Untreated samples were used as reference sample while 18S gene was used as endogenous control (18S forward CCTGCCAGTAGTCATATGCTT, reverse CTCGATCCAATGAACCAAACT).

### 2.8. Mass spectrometry analysis

The identity of the immunopositive band at 75 kDa was analysed through nano ESI mass spectrometry (MS). A protein amount from late gastrula extract, corresponding to 20 µg, was loaded in quadruplicate and separated by SDS/PAGE on 12% gel. The gel was then incubated in a fixative solution (40% methanol, 7% acetic acid) for 1 h at RT and stained with Colloidal Coomassie solution (20% methanol, 16% Brilliant Blue G colloidal concentrate, Sigma) overnight at 4°C. The gel was incubated in a de-staining solution (25% methanol, 10% acetic acid) until the desired de-staining was observed. The band of interest was cut, kept in 5% acetic acid and *in situ* digested with trypsin (protein : protease ratio 20 : 1) upon Cys derivatization with iodoacetamide as described by Tedeschi *et al*. [45] before MS analysis. Peptide separation was achieved on a Thermo Easy-nLC 1000, with a linear gradient from 95% solvent A (2% acetonitrile, 0.1% formic acid) to 30% solvent B (80% acetonitrile, 0.1% formic acid) over 60 min, from 30% to 60% solvent B in 5 min and from 60% to 100% solvent B in 2 min at a constant flow rate of 0.25 µl min^−1^, with a single run time of 75 min. MS data were acquired on a Thermo Q-Exactive–HF, with a data-dependent top 15 method, the survey full scan MS/MS spectra (300–1650 *m/z*) were acquired in the Orbitrap with 60 000 resolution, AGC target 3e6, IT 20 ms. For HCD spectra resolution was set to 15 000, AGC target 1e5, IT 80 ms; normalized collision energy 28 and isolation width of 1.2 *m/z*. Raw label-free MS/MS files from Thermo Xcalibur software (v. 4.1) were analysed using Proteome Discoverer software (v. 2.2, Thermo Fisher Scientific). The minimum required peptide length was set to six amino acids with carbamidomethylation as fixed modification, Met oxidation and Arg/Gln deamidation as variable modifications.

## 3. Results

### 3.1. OvoA mRNA localization during *Paracentrotus lividus* development

**Figure 1.**
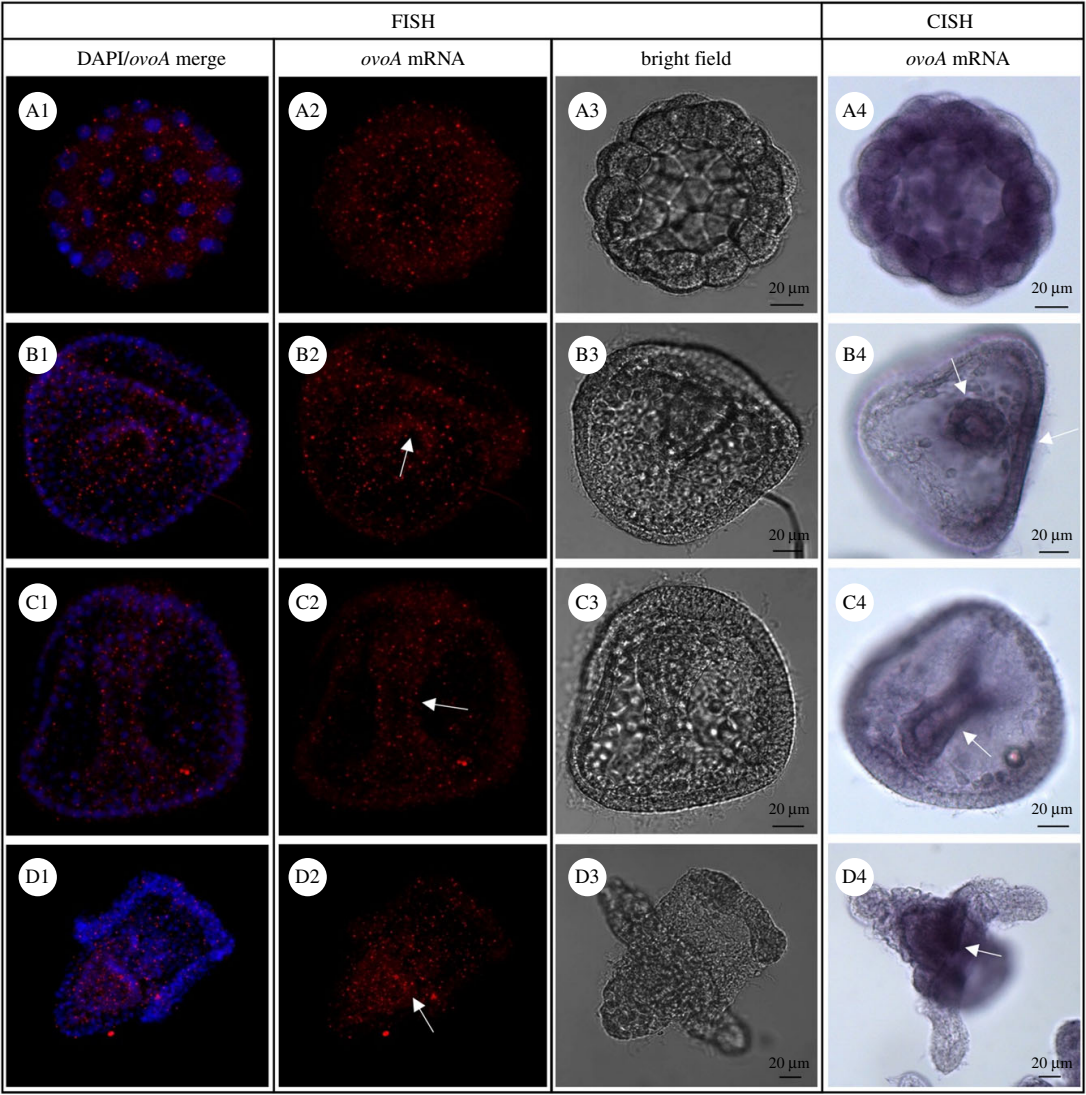
OvoA mRNA localization in *P. lividus* embryos. For FISH detection, ovoA antisense/DAPI merge (on left), ovoA antisense probe signal (middle) and images in bright field (BF, on right) are shown for some representative *P. lividus* developmental stages. (A1–A3) 32-cell stage; (B1–B3) gastrula; (C1–C3) prism stage; (D1–D3) pluteus larva. For CISH detection, bright field images are shown for the same developmental stages (A4–D4). White arrows indicate the cells with stronger signal (for details refer to the text). FISH pictures were taken at confocal microscope (Zeiss LSM 700) at 20× magnification and maximum intensity Z-projections were obtained through ImageJ software. CISH pictures were taken at optical microscope (Zeiss Axio Imager M1) at 20× magnification.

The ovoA mRNA spatial expression in the embryos was investigated by *in situ* hybridization (ISH) experiments performed on *P. lividus* developmental stages. This is the first time the localization of ovoA mRNA and OvoA enzyme has been studied during embryogenesis of an animal model system. Both fluorescent (FISH) and chromogenic (CISH) detection were performed following the incubation of fixed embryos with antisense and sense (negative controls) ovoA probes. FISH experiments in early stages showed a diffuse signal in all blastomeres, without a restricted pattern, until the 32-cell stage (figure 1A2). The experiments performed in gastrula and prism stages showed an ovoA diffuse expression in all cells, although the signal appeared stronger in the archenteron region (figure 1B2, C2). Similarly, in the plutei larvae ovoA mRNA was expressed in all the larva, although a stronger signal was detected in the gut and mesenchyme cells identifiable for their position as skeletogenic cells (figure 1D2). CISH confirmed, to some extent, the results obtained by FISH, despite enhancing some key differences, probably due to the higher sensitivity but lower specificity of the diffusible chromogenic signal. Indeed, CISH results showed a diffuse ovoA expression in all blastomeres at the 32-cell stage (figure 1A4). On the other hand, in the gastrula and prism stages the expression was restricted to the archenteron region, especially in blastopore and oral ectoderm (figure 1B4, C4), although it cannot be excluded that a diffuse ovoA mRNA expression occurred in all other cells as well, due to the presence of weak staining. In plutei larvae there was a strong signal in the gut (figure 1D4), although a weak staining is also present in all the larva. Embryos hybridized with the sense probe, used as negative controls, showed absence of signal both in FISH and CISH experiments (electronic supplementary material, figure S3).

### 3.2. Temporal and spatial expression of ovoA protein

**Figure 2.**
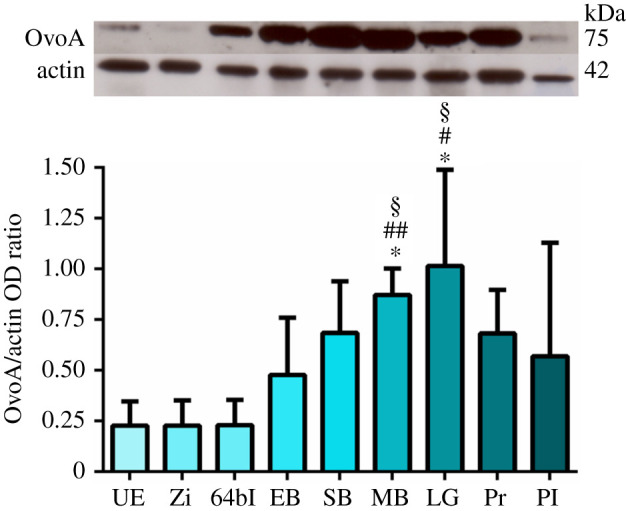
OvoA protein expression during *P. lividus* development. Top: representative western blot (WB) experiment showing the immunopositive bands OvoA (75 kDa) and actin (42 kDa) for all the examined developmental stages, from left to right: unfertilized eggs (UE), zygote (Zi), 64-blastomere stage (64bl), early blastula (EB), swimming blastula (SB), mesenchyme blastula (MB), late gastrula (LG), prism (Pr), pluteus larva (Pl). Bottom: bar-chart showing the OvoA/actin optical densitometry (OD) ratio resulting from WB analyses performed on embryos derived from three different females. Data are presented as means ± s.d. (*n* = 3 biological replicates). *p < 0.05 represents significance compared to UE; #*p* < 0.05 and ##*p* < 0.01 represent significance compared to Zi; §*p* < 0.05 represents significance compared to 64bl.

Different *P. lividus* developmental stages were analysed for OvoA temporal expression through WB analyses. The antibody anti-OvoA recognized a band at the apparent molecular weight of 75 kDa (figure 2), very similar to the theoretical value of the OvoA primary structure (87.9 kDa). The identity of the band at 75 kDa was confirmed by mass spectrometry analysis which revealed the presence of four peptides belonging to OvoA sequence (electronic supplementary material, figure S4).

The intensity of this band, normalized to actin, was monitored from unfertilized eggs to plutei larvae. The results showed high variability among batches. However, a general trend was observed with low OvoA expression in the eggs and early embryos followed by a significant increase at the mesenchyme blastula and late gastrula stages (figure 2; electronic supplementary material, tables S1 and S2). This increase was also detected at swimming blastula and prism stages, although slightly approaching the significance threshold (0.06 < *p*-value < 0.08; electronic supplementary material, table S2).

**Figure 3.**
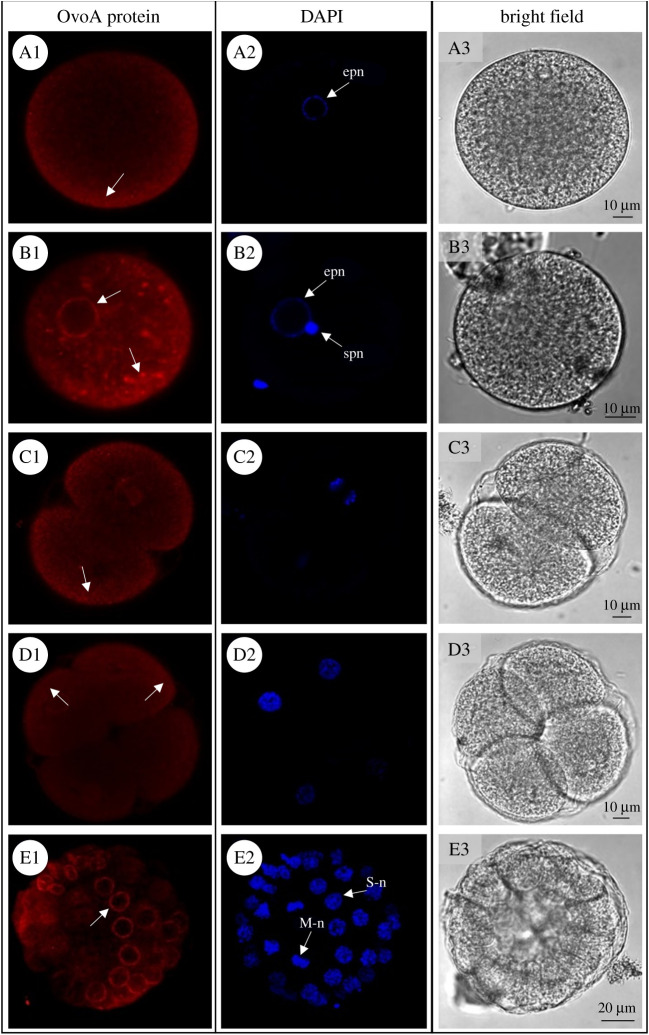
OvoA spatial expression in *P. lividus* early developmental stages. For immunohistochemistry experiments (IHC), OvoA immunofluorescence (in red, left side), nuclei (labelled in blue with DAPI, middle) and images in bright field are shown for: (A1–A3) unfertilized eggs; (B1–B3) fertilized eggs; (C1–C3) 2-cell stage; (D1–D3) 4-cell stage; (E1–E3) 32-cell stage. In OvoA protein panel, white arrows indicate OvoA signal (for details refer to the text). epn = egg pronucleus, spn = sperm pronucleus, S-n = S-phase (interphase) nucleus, M-n = M-phase (mitosis) nucleus. Pictures were taken at confocal microscope (Zeiss LSM 700) at 20× magnification.

**Figure 4.**
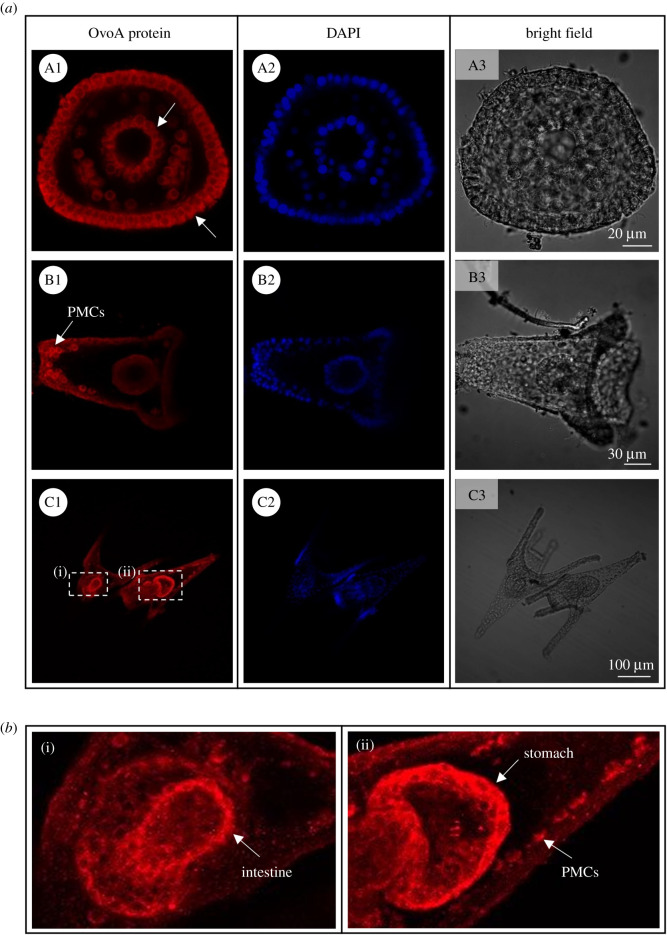
OvoA immunolocalization in *P. lividus* late developmental stages. (*a*) For immunohistochemistry experiments (IHC), OvoA immunofluorescence (in red, left side), nuclei (labelled in blue with DAPI, middle) and images in bright field are shown for: (A1–A3) gastrula stage; (B1–B3) early echinopluteus; (C1–C3) plutei larvae. (*b*) For pluteus stage two zoom insights are reported, showing (i) OvoA signal in intestine, and (ii) stomach and primary mesenchyme cells (PMCs). White arrows indicate OvoA signal (for details refer to the text). Pictures were taken at confocal microscope (Zeiss LSM 700) at 20× magnification.

*Paracentrotus lividus* developmental stages were analysed for OvoA protein spatial expression through IHC experiments. Immunolocalization experiments of the OvoA enzyme performed in unfertilized eggs revealed a faint signal, weakly localized near the plasma membrane (figure 3A1). The signal became stronger in fertilized eggs where several intense spots inside the cell and near the nuclear membrane were detected (figure 3B1). During the first mitotic divisions of the embryo (2- and 4-cell stage) the OvoA immunopositivity was very faint, although a slightly stronger signal was observed near the plasma membrane (figure 3C1 and D1). At the 32-cell stage, a differential immunopositivity was detected in relation to the cell cycle phases. Indeed, a stronger signal was detected in S-phase (interphase) blastomeres compared to those in M-phase (mitosis) (figure 3E1–E2). The IHC experiments at gastrula stage showed the presence of OvoA in the cytoplasm of all cells without a cell-specific localization (figure 4*a*A1). As development proceeded, the signal became gradually restricted to specific cells and tissues. Indeed, in early echinoplutei there was a strong immunopositivity in the skeletogenic primary mesenchyme cells (PMCs) and possibly other mesenchyme cells (figure 4*a*B1). Interestingly, in pluteus larvae, OvoA was clearly localized, as well as in PMCs, stomach and intestine cells (figure 4*a*C1,*b*). Developmental stages probed with pre-immune IgGs, as negative controls, showed an absence of staining (electronic supplementary material, figure S5).

**Figure 5.**
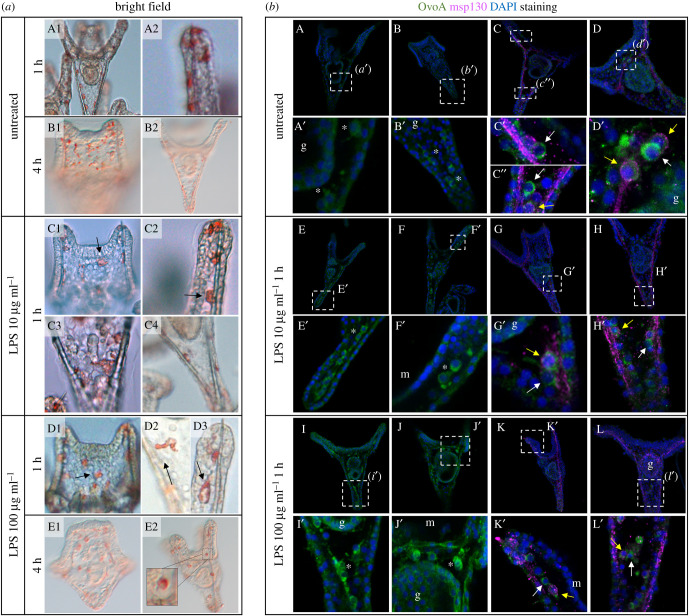
Phenotypic and molecular characterization of *P. lividus* pluteus larvae exposed to LPS treatments. (*a*) Phenotype observation of larvae exposed to various combinations of LPS concentrations and time points, and untreated larvae: (A1–B2) untreated larvae; (C1–C4) larvae after 1 h of exposure to 10 µg ml^−1^ of LPS; (D1–E2) larvae treated with 100 µg ml^−1^ of LPS for 1 h or 4 h (arrows indicate pigment cells inside the larva). (*b*) OvoA (in green) and Msp130 (in purple) immunostaining of untreated larvae (A–D′), and after treatment with 10 (E*–H*′) and 100 µg ml^−1^ (I–*L*′) of LPS for 1 h. g = gut; m = mouth; white asterisks = cells possibly skeletogenic; yellow arrow = skeletogenic OvoA-positive cells; white arrows = non-skeletogenic OvoA-positive cells. Brightness/contrast was adjusted only on the double immunostainings with Msp130, on the zoomed images.

The strong signal of OvoA in the gut, whose associated microbiome plays a pivotal role for the larval immune system [46,47], prompted us to investigate the possible involvement of ovothiol in the larval response against bacterial infection. To this aim, larvae were treated with different concentrations of LPS, the major component of the outer membrane of gram-negative bacteria. At different time points (1 and 4 h), larvae were examined by phenotypic observation, *ovoA* gene expression and protein localization. After treatment with LPS at 10 and 100 µg ml^−1^, pigment cells in the dorsal side of larvae showed a roundish shape, typical of cells that are responding to an infection [48,49] and appeared like migrating from the ectoderm towards the inside of the larva (figure 5*a*C1–E2). By contrast, pigment cells of control larvae were located in the ectodermal epithelium and exhibited a stellated shape, typical of resting cells not responding to any infection (figure 5*a*A1–B2). Treatment with 100 µg ml^−1^ LPS for 1 h induced a slight upregulation of *ovoA* gene expression (electronic supplementary material, figure S6), and an increase of the OvoA immunopositive signal in mesenchyme cells (figure 5*b*I–L′) compared to untreated controls (figure 5*b*A–D′), while 10 µg ml^−1^ LPS seemed to not induce a strong signal increase (figure 5*b*E–H′). Double immunostaining of larvae with antibodies against OvoA and Msp130, a skeletogenic cell marker, revealed the presence of OvoA also in some non-skeletogenic mesenchyme cells (figure 5*b*C′,C″,D′,G′,H′,K′,L′, white arrows), in addition to the skeletogenic ones (figure 5*b*C″,D′,G′,H′,K′,L′, yellow arrows). Interestingly, some of the OvoA positive cells that did not look to be skeletogenic showed extended filopodia and were very close to the gut (figure 5*b*).

### 3.3. OvoA knockdown induces an apoptotic phenotype

**Figure 6.**
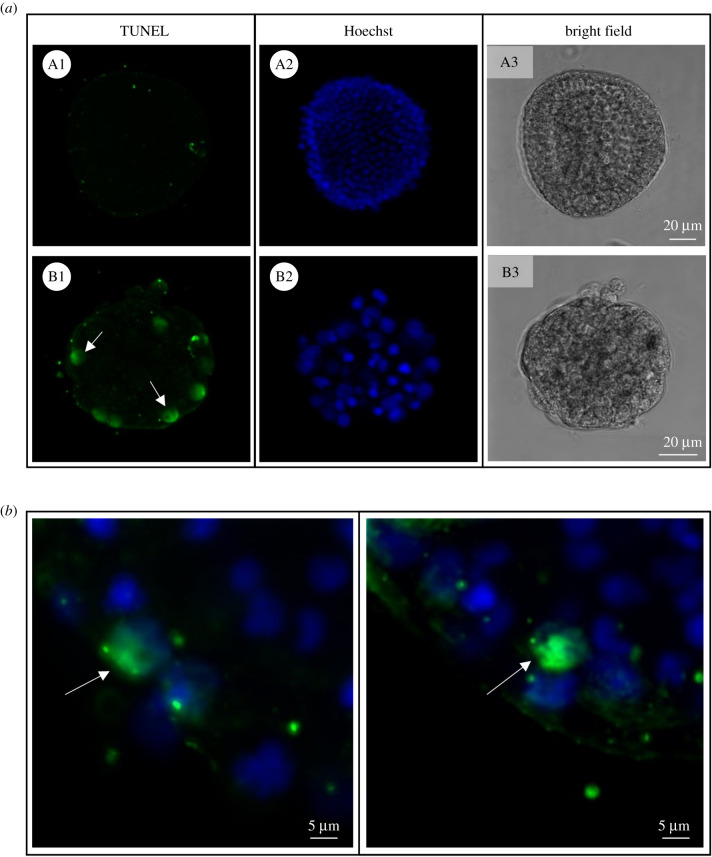
Apoptotic nuclei detection in OvoA knockdown sea urchin embryos. (*a*) TUNEL signal, nuclei staining (Hoechst) and bright field images are shown for both ctrl-MASO (A1–A3) and ovoA-MASO embryos (B1–B3). (*b*) TUNEL/Hoechst merged signal from two additional representative ovoA-MASO embryos. White arrows indicate the TUNEL-positive nuclei.

**Figure 7.**
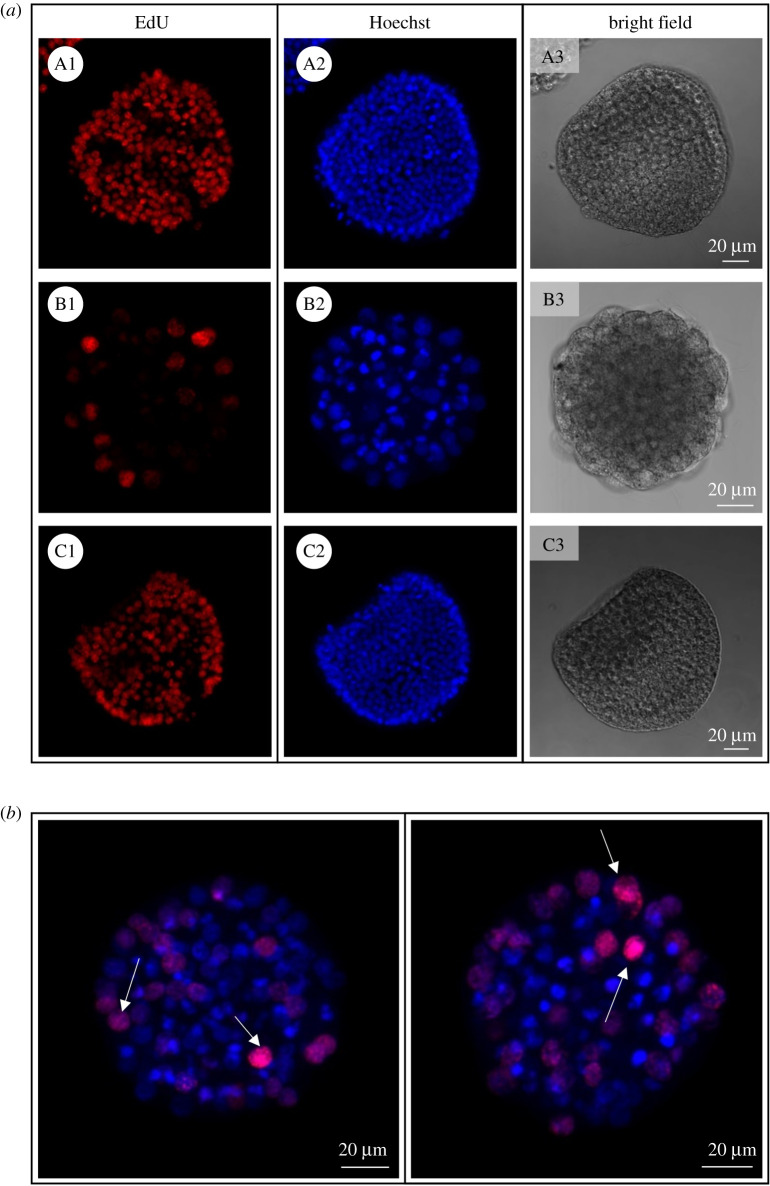
Proliferating nuclei staining in OvoA knockdown sea urchin embryos. (*a*) EdU signal, nuclei staining (Hoechst) and bright field images are shown for embryos injected with ctrl-MASO (A1–A3), ovoA-MASO alone (B1–B3) and co-injected with ovoA synthetic mRNA (C1–C3). (*b*) EdU/Hoechst merged signal from two additional representative ovoA-MASO embryos. White arrows indicate the EdU-positive nuclei. Pictures were taken with a confocal microscope (Zeiss LSM 700) at 20× magnification.

In order to understand the functional relevance of ovothiol during the embryonic development of sea urchin, its formation was perturbed through zygotic microinjection of anti-ovoA MASO (ovoA-MASO), thus blocking the OvoA protein translation. Embryos developed from perturbed zygotes were analysed for phenotype observation and OvoA protein immunopositivity by IHC experiments. The phenotype of perturbed embryos (ovoA-MASO) was compared with phenotype of embryos injected with a control morpholino, which was expected to not cause any malformation (ctrl-MASO). OvoA-MASO embryos showed no malformation until blastula stage, although revealing a slight delay in development compared to ctrl-MASO (electronic supplementary material, figure S7A). At the gastrula stage (18–20 hpf), a high percentage of malformation (80%) was observed in ovoA-MASO embryos, showing an extreme phenotype characterized by the lack of PMCs ingression and archenteron invagination, and resulting in a ‘ball of cells’ with big and fragmented apoptotic-like nuclei (electronic supplementary material, figure S7B). To confirm the occurrence of apoptotic processes, and eventually highlight any effect on cell proliferation, ovoA-MASO embryos were analysed through TUNEL and EdU assays, to detect double-strand DNA breaks and actively replicating DNA, respectively. Results showed the presence of TUNEL positive nuclei in ovoA-MASO embryos, compared to ctrl-MASO, which did not display any apoptotic nucleus (figure 6). Ctrl-MASO embryos treated with DNase were used as positive control of the assay (electronic supplementary material, figure S8). In addition, a decrease in cell proliferation was observed in ovoA-MASO compared to ctrl-MASO embryos, which was rescued by co-injection in zygotes of ovoA synthetic mRNA, obtaining a very low percentage of malformation (2–3%), and the recovery of the wild-type cell proliferation (figure 7).

## 4. Discussion

Previous studies suggested that ovothiol plays a key role in sea urchins acting as a redox regulator to protect eggs from the high oxidative burst at fertilization and developing embryos from environmental stressors [18,28].

Embryonic development is a finely regulated and extremely delicate period in the lifetime of all living organisms, especially for those with external fertilization, such as sea urchins. Sea urchin developing embryos are indeed exposed to a variety of environmentally damaging factors and efficient protection is needed to ensure the progression of the correct developmental programme and ultimately the fitness of the progeny. In this work we investigated the functional role of ovothiol biosynthesis in embryos of the sea urchin *P. lividus*, by assessing the localization of OvoA and perturbing its protein translation, demonstrating a key role for this molecule during development, presumably acting on different biological processes.

### 4.1. Role of ovothiol during early *Paracentrotus lividus* development

The temporal OvoA protein expression pattern from unfertilized eggs to plutei larvae, with a maximum at the blastula/gastrula stages, exhibited a specular trend compared to *ovoA* gene expression data, reported in previous studies, which showed high levels in unfertilized eggs, a strong decrease at early and swimming blastula stages, and then an increase at the pluteus stage [18]. This shift in the transcript and protein expression pattern can be ascribed to the time needed to synthesize the OvoA protein. Indeed, in sea urchins, following fertilization, there is an enhanced activation of cell metabolism, including protein synthesis, being dispensable for the S-phase but necessary for the onset of M-phase and subsequent embryonic cell cycles [50–52]. Thus, it is likely that the previously described decrease in ovoA mRNA in fertilized eggs is followed by a triggered protein translation in the embryo.

In addition, our pioneering protein localization experiments indicate the presence of OvoA near the plasma membrane in unfertilized eggs and its increase in fertilized eggs, thus providing support to Shapiro's theory about a role of ovothiol against the oxidative stress produced by the entry of the spermatozoon into the oocyte [28]. Moreover, the higher expression of OvoA in S-phase (interphase) blastomeres compared to those in M-phase (mitosis) at the 32-cell stage may suggest a correlation of ovothiol biosynthesis with the cell cycle progression. Indeed, fertilization in sea urchins triggers the entry of the eggs, blocked in G1 phase, into the S-phase [52], and ROS are known to play a key role in cell cycle progression, especially for entry into the S-phase [53]. Thus, ovothiol could help to maintain a redox balance, preventing the damaging effects of toxic ROS concentrations produced during the cell cycle, a key step for a normal early development in which the cycle time is extremely short, alternating S and M phases without ‘gap’ [54]. The association of ovothiol formation with key regulators of cell cycle represents an interesting point of further investigation.

Although at the gastrula and prism stages the OvoA protein is expressed in all cells, ISH experiments, especially using chromogenic detection, revealed a stronger signal in the blastopore, archenteron and oral ectoderm. Such accumulation of ovoA mRNA in these tissues is in line with the IHC experiments at the larval stage, revealing a very specific expression pattern of the protein in the digestive tract. Indeed, the accumulation of high mRNA concentrations in blastopore, archenteron and oral ectoderm at gastrula stage could sustain the increased OvoA protein translation in the larval tissues originating from them (e.g. anus, gut and oral cavity, respectively). It is worth noting that the restriction of the protein expression to few specific larval cells and tissues may be responsible for the overall decrease of the protein levels at the pluteus stage, revealed by WB analyses. By contrast, at earlier stages (i.e. blastula and gastrula), the amount of protein showed by WB is higher, probably for its diffuse localization in all cells.

Interestingly, when OvoA protein translation is blocked in the eggs by microinjection of MASO specifically designed against ovoA (ovoA-MASO), the embryos do not develop over the gastrulation process, thus showing a decrease in cell proliferation and the occurrence of an apoptotic-like phenotype. This result might be explained by the presumable link of ovothiol biosynthesis with cell cycle progression in early embryos, because when its biosynthetic pathway is perturbed, embryos are not able to reach the larval stage while undergoing cell cycle arrest with consequent cell death.

Another interesting observation arising from IHC experiments is the restricted OvoA protein expression pattern to the subcellular space surrounding the nuclear membrane, suggesting a possible involvement of ovothiol in the protection of DNA from damaging agents. This hypothesis is in line with the higher OvoA expression in S-phase of the cell cycle, when DNA is actively replicating and eventual stressors could potentially lead to errors in genetic information, which can also be transmitted to the daughter cells. For instance, a strictly localized ovothiol production close to the nuclear membrane could be necessary to protect the embryos from ultraviolet radiation, which can penetrate up to 7–12 m depth into the seawater column, and can eventually affect sea urchin fertilization success, timing of cleavage and development, mainly damaging the DNA by their direct action or through the consequent ROS production [1].

### 4.2. Possible involvement of ovothiol in the larval inflammatory response and skeleton formation

The strong OvoA immunolocalization in the sea urchin gut is intriguing because it represents a key barrier against biotic and abiotic threats the embryos can encounter in the seawater column, like heavy metals, toxins, bacteria/viruses. Since the sea urchin larval gut is an important site of immune defence [49,55], we investigated *ovoA* gene expression and protein localization following infection. We exposed the larvae to a bacterial LPS to induce an immune response, which was indeed detected by the phenotype change of pigment cells: from the resting stellate state, they became rounded and migrated from the ectoderm towards the inside of the larva, such as happens when an immune response is activated [48]. Moreover, non-skeletogenic OvoA positive cells were highlighted, having extended filopodia and being located close to the gut. A coordinated cellular immune response, involving distinct cell types, from pigment to filopodial and ameboid cells, occurs when sea urchin larvae are infected by marine bacteria [49]. The presence of OvoA, and eventually ovothiol formation, in these filopodial non-skeletogenic cells could be related to the ability of ovothiol to counteract ROS overproduction in response to microbial infection [56].

A possible function of ovothiol in the context of an inflammatory response is also supported by the increased expression of the *ovoA* gene after LPS treatment, as revealed by quantitative PCR data, as well as by the increase in the protein expression in mesenchyme cells.

Finally, the clear OvoA positivity in skeletogenic mesenchyme cells suggests that this molecule could be somehow involved in the skeletogenic process. Larval skeletogenesis is a key morphogenetic event, although transient in sea urchin development [57]. The larval skeleton, mainly constituted by a soluble form of CaCO_3_, magnesium calcite [58,59], is essential to protect the digestive organs, and it contributes to the orientation of the arms, necessary for effective swimming and feeding behaviour through the action of the cilia [60–63].

Since skeletogenic cells, or PMCs, and spicules, face to the primary body cavity, whose pH conforms to the external seawater pH, they are directly exposed to any eventual environmental change. Decreases in pH, for example, caused by ocean acidification events, can cause a dysregulation of ion pumps and transporters, challenging the calcifying activity in spicules [64]. Nevertheless, larvae are able to maintain calcification rates also under acidified conditions due to energy allocation mechanisms and the capability of PMCs to counteract an internal acidified environment, protecting the spicules by dissolution [64,65]. Due to the essential role of skeletogenesis for the fitness of the sea urchin offspring and, considering the redox/acid–base properties of ovothiol [66], its biosynthesis in the skeletogenenic cells could help the efficient formation of larval skeleton, by contributing to maintain a redox/acid–base homeostasis inside these cells. This is further suggested by our finding that embryos injected with ovoA-MASO show an extreme phenotype characterized by the lack of PMCs ingression. However, further investigation is necessary in order to clarify these aspects.

## 5. Conclusion

The overall outcomes of this study significantly contribute to our understanding of the biological function of OvoA in marine invertebrates. Due to the pleiotropic properties of ovothiols, including powerful ROS scavenging activities and acid–base regulation properties, the function of OvoA may be essential to ensure correct developmental programme in sea urchins, presumably helping fundamental processes to occur, from cell proliferation to skeleton formation and immune response. This paper reports the first experiments on perturbation of *ovoA* gene function in organismal biology and provides a significant contribution for the understanding of cell and developmental biological processes. Moreover, our findings may also provide inspiration for the discovery of new biological activities of ovothiols in humans, in the light of the growing interest in their beneficial properties.

## Data accessibility

The datasets supporting this article have been uploaded as part of the electronic supplementary material [67].
